# The Janus-Face of Bacteriophages across Human Body Habitats

**DOI:** 10.1371/journal.ppat.1005634

**Published:** 2016-06-23

**Authors:** Adam Wahida, Klaus Ritter, Hans-Peter Horz

**Affiliations:** Division of Virology, Institute of Medical Microbiology, RWTH Aachen University Hospital, Aachen, Germany; Stony Brook University, UNITED STATES

## Introduction

One hundred years after their discovery [1,2], the interest towards the therapeutic use of bacteriophages (briefly: phages) has resurged due to the escalating rise of multi-drug–resistant bacteria [3,4]. However, besides regulatory hurdles, realization of this treatment concept is hampered by efficiency and safety concerns [5]. Conversely, metagenome studies have shown that phages are consistent and dominant members of the human microbiome, suggesting an unknown role of phages in human health and disease [6]. Therefore, the recognition and understanding of phages as common members of the human microbiome may provide the required confidence and pave the way for their targeted medical application, which will go beyond the classical form of phage therapy. Here we provide a holistic perspective regarding the impact of human-associated phages and possible consequences for our well-being.

**Fig 1:**
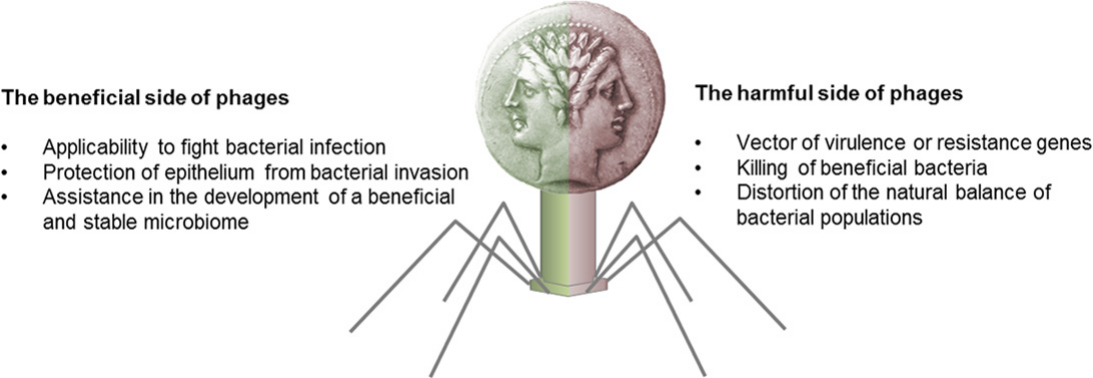
The Janus-Face of phages. Phages are the natural enemies of bacteria, and thus can potentially be used to fight infections caused by multi-drug–resistant strains. However, as mobile vectors, phages can also assist bacteria in providing them virulence or resistance genes. Metagenome studies suggest that some phages may be vital for the development of a beneficial microbiome, while other phages may disturb the bacterial balance, leading to various microbial-driven disorders. The complex interplay between phages and bacteria in humans and the outcome of those interactions is largely unexplored.

The influence of phages is rather complex, because the consequence of infection is not restricted to the immediate host. Any drop-out or altered physiology of the bacterial host can have profound effects on other members within the microbiome as well. Phages are best known for their ability to “arm” bacteria with virulence genes, which converts a benign bacterial strain into a highly pathogenic strain, causing an acute and severe infection [7,8]. Hence, phages are broadly regarded as notorious “providers” of virulence genes, which has conferred upon them a largely negative reputation. However, considering that phages are our permanent companions along with bacteria, the emergence of novel pathogenic bacterial strains is actually a comparably rare event. We believe that from a global perspective, the impacts of phages are rather subtle and mostly in a regulatory and positive way. A stable, healthy microbiome can probably only evolve and be maintained through phage activity. Nonetheless, metagenomic studies have also indicated altered phages populations in dysbiosis. This means that human diseases, characterized by distorted bacterial communities, may ultimately also have phage-driven origins. From this it follows that phages are critical mediators of human health but also of disease, lending them somewhat the character of a “Janus-Face” (Fig 1). It will be one of the greatest challenges of future research to disentangle the dynamic and complex interplays between phages and bacteria. This endeavor is worthwhile, because understanding phage activity in humans (good or bad) can be considered a golden chance for developing strategies that aim at the directed use of phages in favor of human health [9].

The human microbiome is biogeographically structured across the four major habitats: the oral cavity, the gastrointestinal tract, the vagina, and the skin [10,11]. Each habitat is characterized by numerous sub-niches and surfaces harboring a myriad of bacteria, archaea, eukaryotes, and viruses, most of which are phages [9,12–14]. Given the distinctiveness of each body site, phage–bacterial interactions may be quite distinctive as well. However, some general principles may apply, such as the “kill the winner” hypothesis, which argues that the chances of superior (thus growing) bacterial populations encountering phages increases with increasing cell density [15]. This, in turn, initiates the decline of the “winner.” As a consequence, phages aid in sustaining bacterial diversity as they prevent overgrowth of certain bacterial species or strains over others. Clearly, more complex interactions may exist, and a number of models of how bacteria and phages may influence each other have been described recently [6].

## Phages in the Oral Microbiome

The human oral cavity is the prime entry point for viruses and bacteria to access the human body and harbors a highly diverse mixture of transient and resident microbes [11,16]. While earlier studies have suggested that phages do not play an important role in oral microbial ecology [17,18], a different conclusion has been drawn more recently based on metagenomic analyses and on epifluorescence microscopy [19,20]. Virus-like particles have been estimated to make up to 10^8^ particles per milliliter in saliva, of which phages constitute the overwhelming majority. Phage communities remained remarkably stable over months [19,21], but they were found to be quite individual-specific and distinct from those of the gut and other body habitats, which mirrors the distinctiveness of the bacterial communities. A comparably high number of genes encoding for virulence factors were identified, suggesting that phages in the oral cavity may be an important source for pathogenic conditions [19]. For instance, phage-encoded platelet-binding factors in *Streptococcus mitis*-phages, suggest a link of some oral phages with endocarditis [20,22]. A role of phages in periodontal disease has also recently been demonstrated [23,24]. Since periodontal disease is associated with dysbiosis, it may prove to function as a tractable model system to study the involvement of phage populations in mixed infectious diseases [25,26]. We speculate that this research line will ultimately lead to strategies that utilize natural or designed phages for counteracting or preventing periodontal disease.

## Phages in the Gut Microbiome

The human intestinal tract system is undoubtedly the most intensively studied habitat of human-associated microorganisms [27,28], and it is generally recognized that the metabolic activity of a normal endogenous microbiota is vital for human health. Disturbances in microbial community compositions and the subsequently altered microbial physiology have been linked with very diverse and far-reaching disorders, including gastrointestinal diseases, obesity, non-alcoholic fatty liver disease, cancer, brain development, and behavior [29–32]. The tantalizing question here is what role do the endogenous phage populations play? Are they mainly involved in the establishment and maintenance of a beneficial bacterial community? This assumption is warranted, given that phages are ubiquitous in the human gut of healthy individuals [33]. An intriguing argument for the beneficial effects of phages is their presumptive cooperation with the human’s innate immune system, in that harmful bacteria are lysed at the gut/mucosa interface. First evidence has been provided that phages are stably attached to mucosal walls in the gut of animals and humans, and, as such, they may constitute an additional “natural barrier” against potential harmful bacteria [34,35]. The initial establishment of a healthy gut microbiota in newborns may, in part, be due to the activity of protecting phages [36].

Nonetheless, phages may also cause pathological shifts in gut bacterial communities, which may contribute to some (if not all) of the above listed disorders. Inflammatory bowel disease (IBD) is one prominent example in which phage activity and shifts in phage populations have been described [37–39]. Disease-specific shifts in phage populations have been observed for both major forms: Crohn’s disease (CD) and ulcerative colitis (UC) [40]. While phage types differed notably between CD and UC patients, a common feature was the drastic decrease in bacterial richness along with a significant increase of phage populations, compared to healthy individuals. Whether this inversed relationship is the result of induced prophages or strictly lytic phages (domestic or foreign) remains unresolved so far [40].

In any case, the increase of phage population has implications for IBD management. For instance, failure to restore an intact gut microbiome through fecal transplantation from a healthy donor, the concept of which proved successful with *Clostridium difficile* infections, [41] might be due to phage populations interacting with the donor microbes. The consideration of phages as an important component of enigmatic diseases, such as IBD, hails the beginning of future research reinvestigating various gut-related diseases (like obesity, cancer, etc.). Phages may ultimately be used as “prebiotics” to shape microbial communities beneficial to the human gut system [42].

The role of phages in favor of a healthy gut microbiome could also include means of counteracting environmental stressors that cause disturbance, e.g., antibiotics. In fact, metagenomic analysis before and after application of antibiotics in mice indicated a significant increase of antibiotic resistance genes within phage genomes [43]. Furthermore, the number of genes encoding enzymes involved in vitamin, sugar, and lipid metabolism, was also elevated. In addition, processed stool samples with phages from an intestinal flora that had been exposed to an antibiotic substance could confer this resistance to the gut flora of mice that had not been treated with antibiotics [43]. These findings indicate the importance of phages (either lytic or as activated prophages) for sustaining the metabolic and compositional stability of the intestinal flora when exposed to deleterious antibiotics. Phages seem to prevent the most severe consequences of the “pervasive effects” of antibiotics on the human gut microbiota [44]. As a trade-off, however, this mechanism also occasionally fosters the emergence of multi-drug resistant pathogens.

## Phages in the Vaginal Microbiome

It is well known that lactobacilli (especially *Lactobacillus crispatus* and *Lactobacillus jensenii*), referred to as “Döderlein flora” [45], play crucial roles in maintaining a healthy vaginal flora [46]. Bacterial vaginosis (BV) is a microbially driven disorder characterized by the overgrowth of anaerobes, specifically *Gardnerella vaginalis*, following the depletion of the natural lactobacilli population [47]. Among other substances, lactobacilli produce lactic acid through fermentation of dextrose, thereby creating an acidic environment, hostile to many harmful bacteria such as *Neisseria gonorrhoeae* and *G*. *vaginalis* [48,49]. For as yet unknown reasons, lysogenic phages within lactobacilli become activated, lyse their host, and enable pathogenic bacteria such as *G*. *vaginalis* to flourish [50,51]. Since BV occurs more frequently in smokers, substances present in cigarette smoke were suspected to induce the lysogenic phage [52]. In fact, increased concentration of benzo[a]pyrene have been found in the cervix mucosa of smokers [53], and this substance has been identified as a potent inducer of vaginal *Lactobacillus* phages [54]. Increased knowledge of the implication of lactobacilli phages in the pathogenesis of BV might enable, in turn, phage-based strategies for prevention or countermeasures. This would be worthwhile, given that the treatment with metronidazol or clindamycin is the only available therapy with a relatively high recurrence rate [47].

## Phages in the Skin Microbiome

The skin is yet another environment populated with a myriad of different microbial species [55–59]. Prominent members are *Staphylococcus* species and *Propionibacterium acnes*, the latter of which dominates within hair follicles and sebaceous glands and is generally linked with the condition of acne. Consequently, staphylococcal phages and phages against *P*. *acnes* have also been found to be prevalent members of the skin microbiome [60,61]. Unlike most other phages, *P*. *acnes* phages are characterized by a conspicuous lack of genetic diversity [62]. Because of their genome stability along with their relatively broad host range, the suitability of *P*. *acnes* phages for anti-acne treatment has been proposed [62,63]. Given the reduced concerns about phages as topical applications, phage therapy will likely be first realized in the Western world for microbial-driven skin disorders [61].

## Final Remarks

Phages are powerful forces that control bacterial communities, and, as such, they direct the complex relationship between humans and their bacterial companions. Phage activity assists in the constitution of a healthy human microbiome. However, better known, because easier to prove, are their occasional transfer of virulence or antibiotic-resistance genes to bacteria. Differences in diversity or type of phages between healthy and diseased individuals are currently being discerned, but cause and effect have remained largely obscure so far. The analysis of the human virome will therefore continue to be a hot topic of current metagenome research.

In the meantime, on another scientific stage, researchers are desperately looking for alternatives against deadly multi-drug–resistant bacteria. Besides predatory bacteria, such as *Bdellovibrio bacteriovorus* [64] or antimicrobial peptides, [65] phage therapy is one promising alternative. And although conventional phage therapy has proven itself in some Eastern European countries for decades, its breakthrough in the West has yet to come [66]. This is because of a number of technical obstacles that have to be overcome [67–70]. However, development is also slow because of prevailing skepticism. The association of phages with disease rather than with health is more broadly recognized. This is a cognitive bias, which explains the reluctance towards phage therapy and is driven by the counterintuitive character of the concept of phage therapy, namely, to use alien viruses as therapeutics.

What aids in gaining more confidence is the recognition of the natural occurrence of phages in humans, which signifies novel avenues in medicine. Pathological processes will be reinterpreted, attributing phages a potential role in the genesis or prevention of diseases. This knowledge then opens new opportunities to develop targeted therapies, in which phages can be conceived to destroy deleterious bacterial communities or reconstruct the natural bacterial balance. We postulate that from a holistic point of view, phages and humans are friends rather than foes. One hundred years after his discovery of phages, d'Hérelle’s dream of an effective and wide use of phage therapy may, in fact, become a reality but in a much more multifaceted way than could be anticipated at the early days of microbiology.
